# Representation of Female Faculty at US Medical Schools and Success in Obtaining National Institutes of Health Funding, 2008-2018

**DOI:** 10.1001/jamanetworkopen.2021.0388

**Published:** 2021-03-02

**Authors:** Elizabeth Burney Malinzak, Daniel Weikel, Madhav Swaminathan

**Affiliations:** 1 Department of Anesthesiology, Duke University School of Medicine, Durham, North Carolina

## Abstract

This cross-sectional study assesses the representation and success of female faculty at US medical schools and their success in obtaining funding from the National Institutes of Health (NIH) between 2008 and 2018.

## Introduction

Although half of medical school graduates are women, inadequate retention and advancement of women in academia remains concerning. Numerous studies have attempted to identify factors contributing to the “leaky pipeline.” In 2008, the National Institutes of Health (NIH) recognized that more funding was granted to senior scientists. Consequently, the New Investigator policy was modified to enhance success for early-stage investigators. Because early-stage investigators include more women and individuals from minority populations,^1^ the policy was expected to enhance diversity and decrease attrition. In 2019, 34% of NIH grants were awarded to women, up from 28% 10 years earlier.^2^ Studies examining sex and funding since the New Investigator change have shown continued discrepancies.^3,4^ However, at 2 highly funded (HF) medical schools, there was no sex difference in attaining NIH awards.^5,6^ We questioned whether, compared with lowly funded (LF) schools, HF medical schools achieve sex balance in successful grant awarding by having more female faculty overall and by rank. We conducted a descriptive cross-sectional study to test the hypotheses that (1) greater NIH funding is associated with a higher percentage of female faculty and (2) over time, HF schools have better representation of female faculty by academic rank.

## Methods

The 2018 NIH Reporter database was modified to combine funding of associated medical schools with American Association of Medical Colleges (AAMC) schools. The next step was to sort by total funding to determine the 20 HF schools (top half of the first quartile) and 20 LF schools (bottom half of fourth quartile). American Association of Medical Colleges data on sex and rank were retrieved. We conducted 2-sample paired, 2-tailed *t* tests between the HF and LF groups in the mean percentage or the mean percent change of female faculty overall and by rank for complete years from 2008, 2013, and 2018 (years corresponding to the New Investigator policy modification, 5 and 10 years later). Two-sided *P* < .05 were considered significant. An exemption was granted by the Duke University Institutional Review Board because publicly available data were used and the study did not involve the use of human participants or protected health information. The same institutional review board deemed the study exempt from informed patient consent. All data were analyzed using R version 4.0.0 (Foundation for Statistical Computing). The Strengthening the Reporting of Observational Studies in Epidemiology (STROBE) reporting guideline was used for this cross-sectional study.

## Results

There was no difference between the 20 HF and 20 LF schools in the mean percentage of female faculty in 2008 (35.13% vs 33.46%; *P* = .24), 2013 (37.81% vs 36.60%; *P* = .47), or 2018 (41.04% vs 38.25%; *P* = .09). In addition, there was no significant difference in the change in percentage of female faculty (2008 to 2013: 2.69% for HF schools vs 3.19% for LF schools; *P* = .52; 2008 to 2018: 5.92% for HF schools vs 4.78% for LF schools; *P* = .26). However, there was a significant increase from 2008 to 2018 in the mean percentage of female faculty in HF schools (35.13% vs 41.04%; *P* < .001) and LF schools (33.46% vs 38.25%; *P* = .02).

When the data were examined by academic rank, there was no difference between HF and LF schools in the mean percentage of female faculty at the instructor, assistant professor, associate professor, or professor ranks (Table). Compared with the mean percentage change of female faculty, there was no difference between the groups except that HF medical schools had a greater increase in female professors compared with LF medical schools (2008 to 2013: 1.15% vs 0.29%; *P* = .01; 2008 to 2018: 2.17% vs 0.61%; *P* = .007) (Figure).

**Table.** Mean Percentage of Female Faculty by Rank in Medical Schools With High and Low NIH Funding

Rank	Year	Mean %		*P* value
		High NIH funding	Low NIH funding	
Instructor	2008	4.85	3.34	.25
	2013	4.26	2.95	.33
	2018	4.01	2.73	.34
Assistant professor	2008	16.11	18.27	.13
	2013	17.29	20.06	.07
	2018	19.16	21.26	.20
Associate professor	2008	6.84	6.85	.99
	2013	7.67	8.20	.56
	2018	8.19	8.85	.57
Professor	2008	5.09	4.50	.48
	2013	6.24	4.79	.14
	2018	7.26	5.11	.06
Other rank^a^	2008	2.23	0.5	.07
	2013	2.35	0.6	.11
	2018	2.42	0.3	.05
All ranks	2008	35.13	33.46	.24
	2013	37.81	36.60	.47
	2018	41.04	38.25	.09

Abbreviation: NIH, National Institutes of Health.

^a^ Per the American Association of Medical Colleges, the other rank category includes all faculty whose positions do not conform to one of the 4 standard ranks (professor, associate professor, assistant professor, or instructor).

**Figure.**
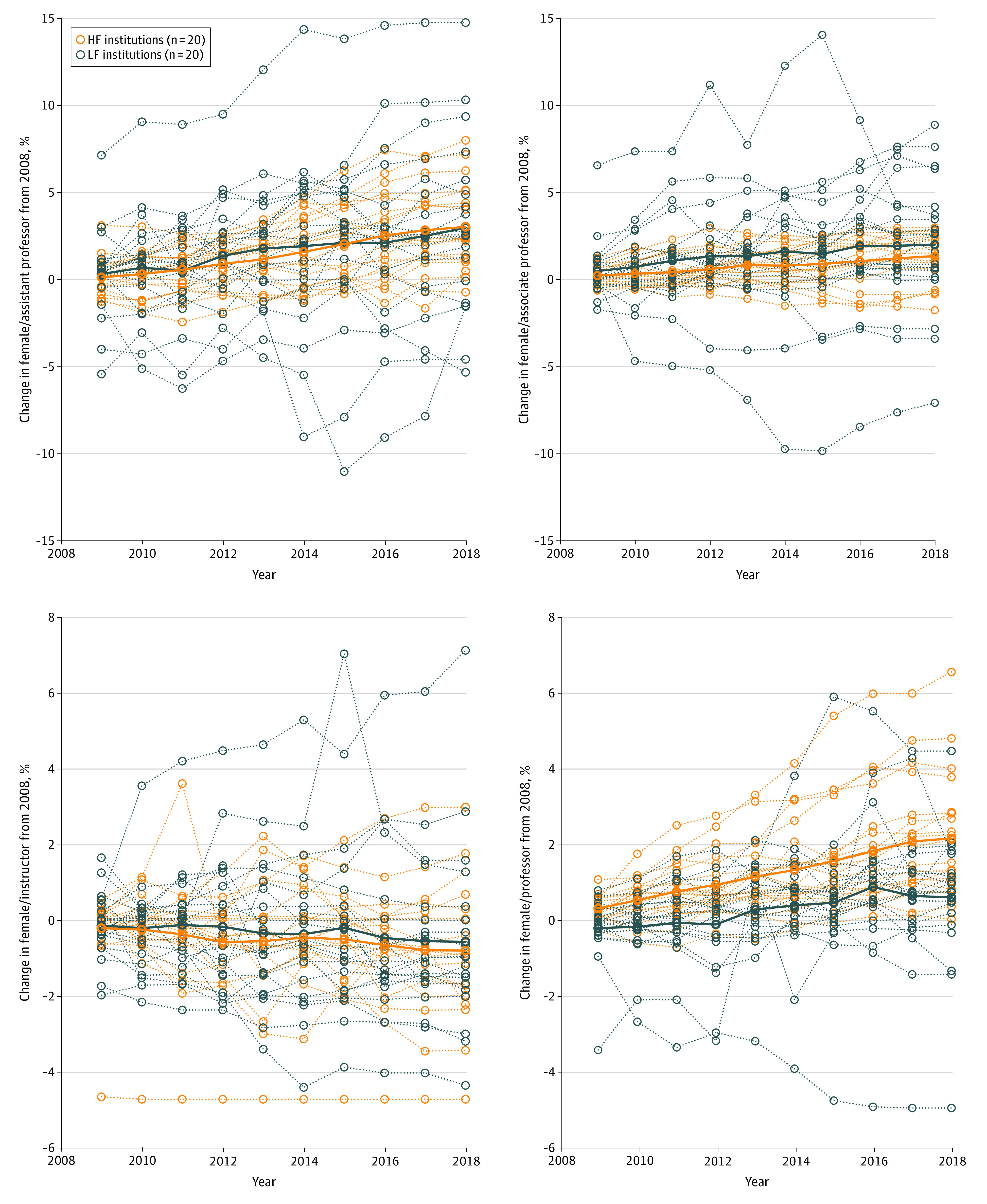
Change in the Mean Percentage of Female Faculty by Rank in Medical Schools With High Funding (HF) and Low Funding (LF) From the National Institutes of Health (NIH) Each panel shows the change in the mean percentage of female faculty at each rank (instructor, assistant professor, associate professor, and professor) over a 10-year period (from 2008 to 2018). Data for each medical school are plotted with dashed lines; the mean of each group is represented with a solid line.

## Discussion

We could not confirm that greater NIH funding was associated with more female faculty or that HF schools had better representation of female faculty over time. For all schools, the change in female faculty was modest (5.92% for HF schools; 4.78% for LF schools). Considering that medical school matriculations for women increased by 30% in the same period, this course is not sustainable. Our analysis suggests that funding is associated only with the professor level at HF schools, indicating more support is needed for midcareer investigators in addition to early-stage investigators. Despite efforts to enhance funding diversity, the pool has remained unchanged. Limitations of our study include the mismatch of medical schools between the NIH and American Association of Medical Colleges databases and the lack of data about nonbinary faculty. Unfortunately, attrition of women in academia is likely to continue unless aggressive efforts are implemented. The NIH launched the Next Generation Researchers Initiative in 2017, and investigation of this policy and others will be necessary.
